# Potential of miRNAs in urinary extracellular vesicles for management of active surveillance in prostate cancer patients

**DOI:** 10.1038/s41416-021-01598-1

**Published:** 2021-11-22

**Authors:** Manuel Ramirez-Garrastacho, Viktor Berge, Aija Linē, Alicia Llorente

**Affiliations:** 1 Department of Molecular Cell Biology, Institute for Cancer Research, Oslo University Hospital, Oslo, Norway; 2 Department of Urology, Oslo University Hospital, Oslo, Norway; 3 Institute of Clinical Medicine, University of Oslo, Oslo, Norway; 4 Latvian Biomedical Research and Study Centre, Riga, Latvia; 5 Department for Mechanical, Electronics and Chemical Engineering, Oslo Metropolitan University, Oslo, Norway

**Keywords:** Biomarkers, Prostate cancer

## Abstract

**Background:**

Active surveillance is an alternative to radical treatment for patients with low-risk prostate cancer, which could also benefit some patients with intermediate risk. We have investigated the use of miRNA in urinary extracellular vesicles to stratify these patients.

**Methods:**

NGS was performed to profile the miRNAs from small urinary extracellular vesicles in a cohort of 70 patients with prostate cancer ISUP Grade 1, 2 or 3. The most promising candidates were then analysed by RT-qPCR in a new cohort of 60 patients.

**Results:**

NGS analysis identified nine miRNAs differentially expressed in at least one of the comparisons. The largest differences were found with miR-1290 (Grade 3 vs. 1), miR-320a-3p (Grade 3 vs. 2) and miR-155-5p (Grade 2 vs. 1). Combinations of 2–3 miRNAs were able to differentiate between two ISUP grades with an AUC 0.79–0.88. RT-qPCR analysis showed a similar trend for miR-186-5p and miR-30e-5p to separate Grade 3 from 2, and miR-320a-3p to separate Grade 2 from 1.

**Conclusions:**

Using NGS, we have identified several miRNAs that discriminate between prostate cancer patients with ISUP Grades 1, 2 and 3. Moreover, miR-186-5p, miR-320a-3p and miR-30e-5p showed a similar behaviour in an independent cohort using an alternative analytical method. Our results show that miRNAs from urinary vesicles can be potentially useful as liquid biopsies for active surveillance.

## Background

Prostate cancer was the second most diagnosed cancer in men in 2020 [1]. Many prostate cancer patients have tumours that are confined to the prostate, which can be cured by radical treatment such as prostatectomy or radiotherapy. Treatments that involve radical therapies entail a reduced risk of disease progression to metastatic disease but may have several adverse secondary effects, such as incontinence, erectile dysfunction and reduced bowel function [2]. A significant number of patients have indolent tumours that will not pose a threat to their lives and do not require radical treatment. In the last two decades, the use of active surveillance (AS) has emerged as an alternative to radical therapies for these patients [3].

AS consists of regular checkups and treatment of the patient only in case of disease progression. The implementation of AS requires finding parameters that clearly distinguish patients that truly have indolent disease. The inclusion criteria for AS programs differ between institutions [4–6], but there is an emerging consensus that it should be the preferred option for most patients with Gleason score (GS) 6. This is based on several studies reporting a risk of clinical progression of 0.2–5% at 15 years and 0.5–3% cancer-specific mortality at 10–15 years [3, 7]. Moreover, the Prostate Cancer Research International AS (PRIAS) study also showed that AS is a safe option for men with low-risk prostate cancer (considered as GS ≤ 6, prostate-specific antigen (PSA) ≤10 ng/ml, and clinical stage not higher than T2c) [8].

There is less agreement about the optimal treatment for prostate cancer patients with intermediate risk of progression (GS 7), who are often treated by radical therapy. Importantly, it has been shown that patients with GS 3 + 4 do not have the same prognosis as patients with GS 4 + 3. This observation led to a novel prostate cancer grading by the International Society of Urological Pathology (ISUP) that splits GS 7 into a favourable low-intermediate risk (ISUP Grade 2) and an unfavourable high-intermediate-risk (ISUP Grade 3) group [9]. It has been reported that, in some situations, men with biopsies in Grades 1 and 2 have similar biochemical recurrence-free survival rates after radical treatment [10]. Therefore, AS seems to be safe for low-risk and some intermediate-risk patients, and more precise stratification tools for these patients are necessary to provide optimal treatment. In this context, liquid biopsies are very relevant for AS because minimally invasive molecular tests that can be serially repeated are very convenient for the selection and follow-up of AS patients. Moreover, prostate cancer tumours are heterogeneous, and liquid biopsies may better reflect the tumour heterogeneity than tissue biopsies.

The most commonly analysed components in  liquid biopsies have been circulating tumour cells and circulating tumour DNA and proteins [11]. Recently, extracellular vesicles (EVs) shed from tumours into biofluids have also started to be analysed in  liquid biopsies [12, 13]. EVs are released by cells by two main mechanisms, including budding from the plasma membrane and extracellular release of multivesicular bodies (MVB) [14]. The analysis of EV-derived biomarkers presents several advantages when compared with the analysis of other components of liquid biopsies. EVs are found in most human biofluids and have a diverse molecular cargo (proteins, nucleic acids, such as mRNA and miRNA, lipids, metabolites) that represents the status of the tissue of origin [15]. Besides, EV molecules may be more stable than free circulating biomarkers because they are encapsulated inside a lipid bilayer [16]. Currently, urine is the biofluid of choice in many studies that aim at identifying new biomarkers for prostate cancer (for recent reviews, see refs. [17–20]). Urine collection is an easy and non-invasive procedure where large amounts of sample can frequently be obtained [21], and prostate-derived EVs get into urine when it flows through the prostatic urethra.

In the last years, several research groups have analysed the usefulness of urinary EV molecules as biomarkers for prostate cancer [18, 19, 22, 23]. Some studies have focused on mRNAs, like the IntelliScore [24] or the combination of PCA3 and PCGEM1 [25], while others have analysed proteins, such as the seven protein panel described by Sequeiros et al. [26] or the combination of CD9, CD63 and PSA [27]. miRNAs are also present in EVs and could be used as cancer biomarkers [28]. miRNAs are small single-stranded non-coding RNA molecules that affect gene expression through the degradation of specific mRNAs. They have been related to cancer both as suppressors and promoters for most types of tumours [29]. miRNAs have a series of advantages that makes them ideal candidates as cancer biomarkers [30]. They are easy to analyse in non-invasive liquid biopsies, show high tissue specificity and can be used as multimarker models for diagnosis, prognosis and evaluation of treatment response.

Several studies have reported urinary miRNAs which could act as biomarkers for prostate cancer. For example, the levels of hsv1-miR-H18 and hsv2-miR-H9-5p [31] and miR-148a and miR-375 [32] have been reported to be better predictors of the disease than serum PSA when comparing prostate cancer patients with healthy individuals and/or men with benign prostate hyperplasia (BPH). Other studies have looked specifically at miRNAs isolated from EVs. Several studies have compared men with prostate cancer to healthy men and identified miRNAs than separate the two groups such as miR-375, miR-451a, miR-486-3p and miR-486-5p [33], miR-107 and miR-574 [34], miR-21, miR-375 and let-7c [35], miR-196a-5p and miR-501-3p [36] or miR-19b [37]. In addition, the potential of combining different types of urinary EV cargo, including two miRNAs, seven mRNAs and the non-coding RNA PCA3, to separate the two groups has recently been shown [38]. Other studies have compared prostate cancer patients to BPH patients and found potential miRNA biomarkers, such as miR-145 [39] and a 3 miRNA ratio model based on miR-222-3p, miR-24-3p and miR-30c-5p [40]. Finally, miRNAs from urinary EVs have also been used to discriminate between patients with different GS. When comparing patients with GS 6, 7 and 8, the level of miR-2909 was shown to be higher in more aggressive prostate tumours [41], and 6 miRNAs have been reported to be differentially expressed in patients with GS ≥ 9 [42].

In this study, we have used next-generation sequencing (NGS) to identify new EV-derived miRNA biomarkers able to classify prostate cancer patients with 1–3 ISUP grades. The most promising miRNAs were then analysed by reverse-transcription quantitative PCR (RT-qPCR) in an independent cohort. To our knowledge, this is the first time that miRNAs in urinary EVs are used to distinguish patients previously included in the same intermediate-risk group based on having GS 7.

## Methods

### Urine samples

Urine samples were collected during the morning (not first-void) from patients with biopsy ISUP Grades 1, 2 and 3 (done by magnetic resonance imaging-ultrasound fusion using the Koelis platform [43]) scheduled for radical prostatectomy within 1 week. The two cohorts used in the study (70 samples for NGS analysis and 60 samples for RT-qPCR analysis) were collected sequentially. The clinical characteristics of both cohorts are presented in Supplementary Tables 1 and 2. The pH of the urine samples and the presence of leucocytes, nitrites, protein, glucose, ketones and blood in urine were analysed with a Combur-Test strip in an Urysys 1100 urine analyzer (Roche Diagnostics, Basel, Switzerland) to get additional information about the health status of the patients (Supplementary Tables 1 and 2). The collection of urine samples was approved by the Norwegian Regional Committees for Medical and Health Research Ethics and the participants gave informed written consent.

### Isolation of urinary small EVs

EVs were isolated from fresh urine samples by differential centrifugation as previously described [44]. Briefly, urine samples (between 25 and 150 ml) were centrifuged at 2000×*g* for 15 min at room temperature (RT) to remove cells and cell debris, and then at 10,000×*g* for 30 min at RT to separate large particles. The resulting supernatant was centrifuged at 100,000×*g* for 70 min at RT in a Ti70 fixed-angle rotor (31,000 RPM, *k* factor = 224) in 31-ml thick wall polypropylene tubes (Ref. 355642, Beckmann Coulter, Indianapolis, IN, USA). The pellet was washed with 20 ml phosphate-buffered saline (PBS) and centrifuged again at 100,000×*g* for 70 min at 4 °C in a Ti70 rotor. The pellet was then resuspended in 10 ml PBS, vortexed, filtered through a 200-nm pore Supor syringe filter (Pall, Port Washington, NY, USA) and finally the solution was centrifuged at 100,000×*g* for 70 min at 4 °C in a swinging bucket SW40 rotor (24,000 RPM, *k* factor = 379) in 10-ml polypropylene tubes (Ref. 358210, Beckman Coulter, Indianapolis, IN, USA). Most of the supernatant was then removed, leaving 50–100 μL PBS at the bottom, which was used to resuspend the pellet. All the ultracentrifugations were done in an Optima centrifuge L-90K (Beckmann Coulter, Indianapolis, IN, USA) using maximum acceleration and deceleration. The amount of sample was estimated by measuring the concentration of total protein in the EV pellet and the EV counts with a Zetasizer Nano ZS (Malvern Panalytical, Malvern, UK). Then, the samples were stored at –80 °C until further use.

### Protein measurements

The amount of total protein in EV samples was determined either with a bicinchoninic acid (BCA) assay kit (Thermo Fisher Scientific, Waltham, MA, USA) using bovine serum albumin as standard protein or with a Qubit protein assay (Thermo Fisher Scientific, Waltham, MA, USA). Both methods were performed according to the manufacturer’s instructions.

### SDS-PAGE and western blot

EV samples isolated from equivalent volumes of urine were mixed with Laemmli Sample Buffer (Bio-Rad, Hercules, CA, USA) and subjected to SDS-PAGE using the Mini-Protean system (Bio-Rad, Hercules, CA, USA). Proteins were transferred to a PVDF membrane, which was then blocked with 5% skim milk powder (Sigma-Aldrich, St. Louis, MO, USA) for 1 h. Membranes were incubated overnight with primary antibodies at 4 °C. The next day, the membranes were incubated with horseradish peroxidase-conjugated secondary antibodies (Jackson Immunoresearch, Ely, UK) in 5% skim milk powder for 1 h. Protein bands were detected with the SuperSignal West Dura kit (Thermo Fisher Scientific, Waltham, MA, USA).

The following primary antibodies were used: CD9 (ab92726, Abcam, Cambridge, UK), CD63 (H5C6, DSHB, Iowa City, IA, USA), CD81 (ANC-302-020, Nordic BioSite, Stockholm, Sweden), Alix (sc-53540, Santa Cruz, Dallas, TX, USA), Syntenin (ab133267, Abcam, Cambridge, UK), Tsg101 (612697, BD Biosciences, Franklin Lakes, NJ, USA), Ezrin (E8897, Sigma-Aldrich, St. Louis, MO, USA), Annexin 2 (610068, BD Biosciences, Franklin Lakes, NJ, USA), Uromodulin (sc-20631, Santa Cruz Biotechnology, Dallas, TX, USA), Albumin (MAB1455-SP, R&D Systems, Abingdon, UK).

### Nanosight analysis

A Nanosight NS500 instrument (Malvern Panalytical, Malvern, UK) was used to measure the size and number of particles in the 10,000×*g* and 100,000×*g* pellets. The samples were diluted to the optimal working concentration of the instrument (2 × 10^8^ to 1 × 10^9^ particles per ml) with PBS (filtered through a 0.02-µm Anotop 25 filter), and vortexed for 1 min. The samples were then measured and five videos of 60 sec were acquired for every sample. Videos were subsequently analysed with the NTA 3.4 software, which identifies and tracks the centre of each particle under Brownian motion to measure the average distance the particles move on a frame-by-frame basis.

### RNA extraction from urinary EVs

Urinary EVs (~2–4 µg total protein per sample) were treated with 1 mg/ml Proteinase K (Sigma-Aldrich, St. Louis, MO, USA) for 60 min and 10 ng/µl RNAse A (Roche Diagnostics, Basel, Switzerland) for 15 min to remove the RNA that was bound on the surface of EVs or free in the sample solution. Total RNA was then extracted from the vesicles using a miRNeasy Micro Kit (Qiagen, Hilden, Germany) following the manufacturer’s instructions for on-column digestion with RNAse-free DNase. Samples were eluted in 30 µL of RNAse-free water, and 1 µL of RNA was then run in an Agilent 2100 Bioanalyzer using an RNA 6000 Pico Kit (Agilent Technologies, Santa Clara, CA, USA) to detect potential degradation and ribosomal RNA contamination.

### Next-generation sequencing (NGS)

NGS was performed at the Genomics Core Facility (Oslo University Hospital, Norway). Small RNA libraries were prepared using the CleanTag Small RNA library prep kit (TriLink Biotechnologies, San Diego, CA, USA) with index primers (Illumina, San Diego, CA, USA, sets 1–4). Total RNA was ligated to 3’- and 5’-RNA adapters, and reversely transcribed to generate cDNA libraries for each sample. Libraries were PCR amplified, pooled and size selected using acrylamide gel (6%, Novex TBE Gel, Thermo Fisher Scientific, Waltham, MA, USA) purification. Small RNA libraries were sequenced using a NextSeq500 (Illumina) instrument using HighOutput kit v2.5. Fastq files for each sample were processed with Cutadapt [45] to remove adaptors and then analysed using the software package miRDeep2 [46] to map the sequencing reads to the human genome (hg19) and identify miRNAs.

### Reverse-transcription quantitative PCR (RT-qPCR)

cDNA from EV-derived small RNAs was synthesised using miRCURY LNA RT kit (Qiagen, Hilden, Germany) following the manufacturer’s specifications. The compatible miRCURY LNA SYBR Green PCR kit was used for real-time qPCR amplification using a CFX Connect Real-Time PCR Detection System (Bio-Rad, Hercules, CA, USA) with the following conditions: 95 °C for 5 min, thereafter 45 amplification cycles at 95 °C for 10 s, and 55 °C for 20 s and 72 °C for 20 s. For all miRNAs, pre-designed miRCURY LNA compatible primers were acquired from Qiagen: miR-1290 (YP02118634), miR-320b (YP02119299), miR-1246 (YP00205630), miR-320a-3p (YP00206042), miR-186-5p (YP00206053), miR-30e-5p (YP00204714), miR-155-5p (YP00204308), miR-99b-5p (YP00205983).

### Statistical analysis

All the statistical analyses were performed in R 4.0.3 [47]. NGS data were analysed using the DESeq2 package to find differentially expressed miRNAs between the groups (code available upon request) [48]. In the analysis, only miRNAs with at least ten counts in at least ten samples in one of the groups were included. Reads were normalised using the DESeq2 mean of ratios method (see package documentation). miRNAs were considered differentially expressed if they showed an adjusted *P* value lower than 0.05 (using the Wald test available in the DESeq2 package and the Benjamini–Hochberg correction for multiple testing). Receiver-operating characteristic (ROC) curves and area under the curve (AUC) calculations were done using the pROC package [49]. The least absolute-shrinkage and selection operator (Lasso) analysis was performed using the glmnet package [50] and repeated fivefold cross-validation analysis was done with the caret package using five repetitions [51]. For analysis of RT-qPCR data, the comparative *Cq* method was used, using miR-99b-5p for normalisation.

## Results

### Isolation of urinary EVs

**Fig. 1:**
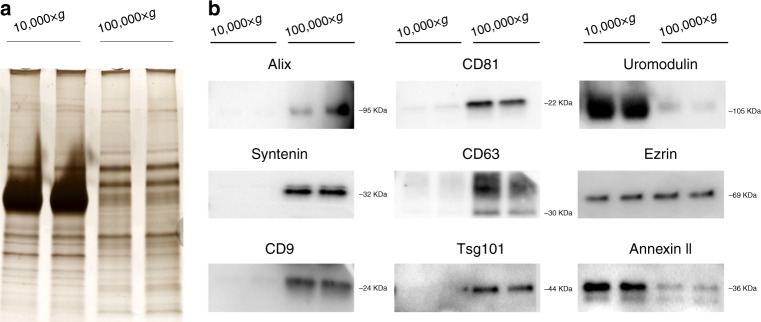
Protein analysis of the 10,000×*g* and 100,000×*g* pellets. Urine samples were first sequentially centrifuged at 2000×*g* and 10,000×*g*. Then the 10,000×*g* pellet was washed with 20 ml PBS and pelleted again at the same speed, while the supernatant was centrifuged at 100,000×*g*, washed twice with PBS and pelleted again at 100,000×*g*. Both pellets were resuspended in 100 µl PBS and the same volume of sample was analysed by silver staining (**a**) and western blot (**b**).

EVs were separated from urine using sequential centrifugation as previously described [52]. This protocol includes a 2000×*g* centrifugation to remove cells and large debris and/or molecular complexes, a 10,000×*g* centrifugation to separate large EVs and an ultracentrifugation step at 100,000×*g* to pellet the small EVs. The 10,000×*g* and the 100,000×*g* pellets obtained from similar volumes of urine were then compared. Silver staining (Fig. 1a) and western blotting of selected proteins (Fig. 1b) showed that the protein profile of the two pellets is different. As shown in Fig. 1a, there was a very abundant protein with a molecular weight of 80-100KDa in the 10,000×*g* pellet. This is in the size range of uromodulin (also known as Tamm-Horsfall protein; THP) the most abundant protein in human urine in normal conditions [53]. Western blot analysis supported the idea that the strong band observed in silver-stained gels is uromodulin because the amount of this protein is also remarkably higher in the 10,000×*g* than in the 100,000×*g* pellet (Fig. 1b). We also observed that the relative amount of total protein associated with the 10,000×*g* and 100,000×*g* pellets showed a great variability from sample to sample (Supplementary Fig. 1A), which could be explained by different amounts of uromodulin in the 10,000×*g* pellet of urine samples from different individuals. However, other soluble proteins in urine may also contribute to the strong band observed in the silver-stained gels. For example, albumin was also detected mainly in the 10,000×*g* pellet (data not shown).

Further analysis of the two pellets by western blot showed that the 100,000×*g* pellet contains higher amounts of several proteins commonly associated to small vesicles originating from MVBs, such as Alix, Tsg101, syntenin and the tetraspanins CD9, CD63 and CD81 [54] (Fig. 1b). Annexin II and ezrin, which are known to be present in both small and large EVs [55], were detected in both fractions (Fig. 1b). A Nanosight analysis showed large differences in the particle number and size distribution of the two pellets (Supplementary Fig. 1B). The number of particles was 10–15 times higher in the 100,000×*g* pellet, which contained particles with a lower mean and mode diameter size than the 10,000×*g* pellet. The 100,000×*g* pellet had been also previously characterised in our group by electron microscopy and it contained EVs with typical morphology which were labelled with CD63 [44]. We decided to continue only with the 100,000×*g* pellet for the miRNA analysis because it more and more homogeneous material.

### Analysis of miRNA in urinary EVs from prostate cancer patients by NGS

The aim of this study was to identify novel miRNAs in small urinary EVs that could be used to differentiate prostate cancer patients based on their tumour grade. We, therefore, performed a discovery study using NGS analysis. Urinary EVs were isolated from 70 prostate cancer patients that were stratified into three groups based on their biopsy ISUP grade: 23 patients had Grade 1 (GS 6), 23 patients had Grade 2 (GS 3 + 4) and 24 patients had Grade 3 (GS 4 + 3). Patient’s data are provided in Supplementary Table 1. For each patient, RNAs were extracted from an amount of EVs equivalent to 3–4 µg of total protein (corresponding approximately to 20 to 60 ml of urine depending on the patient). Prior to RNA extraction, samples were treated with Proteinase K and RNAse to degrade proteins and other RNAs that were not present in the EV lumen. RNAs were then extracted using the miRNeasy Micro Kit. We assessed the quality of these samples using a Bioanalyzer, which showed that the samples had similar profiles and only contained RNAs of less than 200 nucleotides (Supplementary Fig. 2). Following adapter ligation and amplification, the samples were size selected using 6% polyacrylamide gel electrophoresis and subjected to NGS using Illumina high-throughput RNA sequencing technology. An average of 11.6 million raw reads was obtained from each of the RNA sequencing libraries generated. Approximately 54% of them (6.3 million reads) were mapped to known human RNA species, and among them, an average of 33% was identified as miRNAs when compared with the sequences included in miRBase [56]. The most abundant miRNAs were miR-10b-5p, miR-30a-5p, miR-10a-5p, let-7b-5p and miR-26a-5p, which is in concordance with what has been previously found by our group [36]. Two of the samples from Grade 1 were discarded because of their low number of reads (<200,000 reads mapped to miRNAs).

**Fig. 2:**
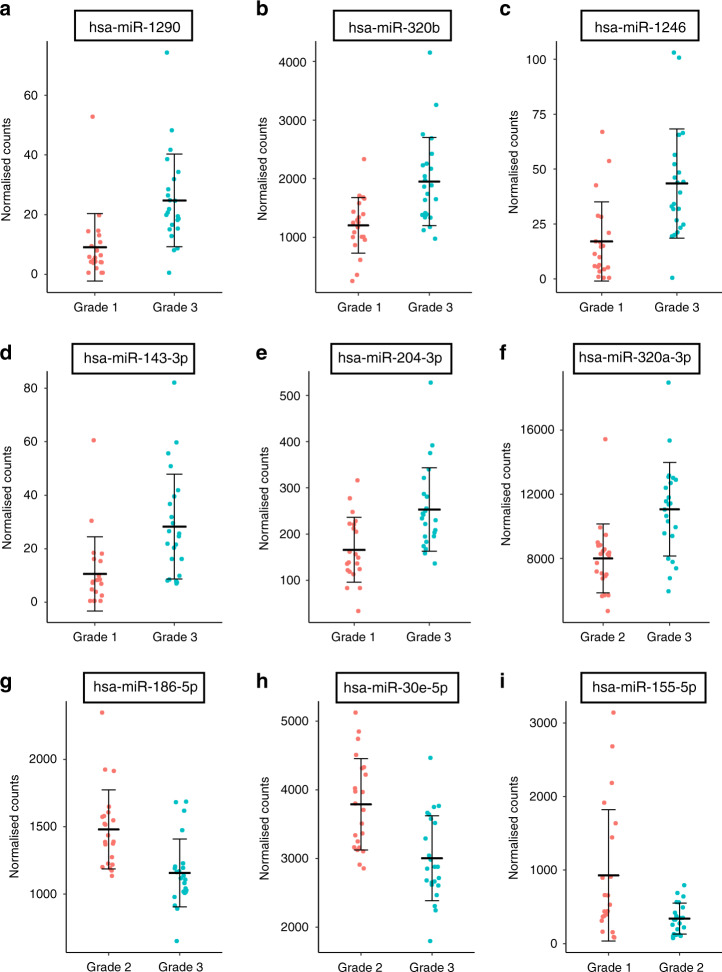
NGS -identified miRNAs in urinary EVs of prostate cancer patients showing differential expression between ISUP grades. The plots show the normalised counts of miRNAs whose expression was significantly changed between Grade 3 and Grade 1 (**a**–**e**), Grade 3 and Grade 2 (**f**–**h**) and Grade 2 and Grade 1 (**i**). Central bars represent the mean and whiskers represent the standard deviation of all the samples.

Each grade group was then compared against each of the other two grades. In addition, Grade 1 was compared to Grades 2 and 3 together, and Grade 3 to Grades 1 and 2 together (see Supplementary File 1 for the results of all the identified miRNAs in each comparison). miRNAs that did not have at least ten counts in at least ten samples in one of the groups were filtered out, leaving between 317 and 347 miRNAs per comparison. In total, nine miRNAs were found to be differently expressed when an adjusted *P* value lower than 0.05 was used as selection criteria (Table 1 and Fig. 2). Six of these miRNAs were detected in all the samples, while miR-1246, miR-1290 and miR-143-3p were not expressed in 4, 5 and 6 of the 68 samples, respectively. The largest number of differentially expressed miRNAs was found between Grades 3 and 1, where five miRNAs were upregulated in patients with Grade 3: miR-1290, miR-320b, miR-1246, miR-204-3p and miR-143-3p (Table 1 and Fig. 2a–e). The increase in the expression level ranged from 1.5 to almost threefold. When comparing Grades 3 and 2, miR-320a-3p was significantly increased (Fig. 2f) and miR-186-5p and miR-30e-5p decreased in Grade 3 (Fig. 2g, h). These three miRNAs showed a small variation in the expression level, which was between 1.26 and 1.38 times. Only one miRNA showed a statistically significant difference when Grades 2 and 1 were compared: miR-155-5p (Fig. 2i), which was decreased almost threefold in Grade 2 samples. Grouping Grades 2 and 3 together and comparing them against Grade 1 did not show any differentially expressed miRNA using the selected criteria. The lowest p-value corresponded to miR-155-5p (adjusted *P* value 0.1), which was upregulated in Grade 1. This was also the only miRNA differentially expressed between Grades 1 and 2. When Grades 1 and 2 together were compared to Grade 3, miR-320a-3p showed a significant increase in patients with Grade 3 (Table 1). Another three miRNAs which had been found in other comparisons (miR-1290, miR-204-3p and miR-30e-5p) showed an adjusted *P* value lower than 0.1 (Supplementary File 1). As discussed later, all the differentially expressed miRNAs found in this analysis had been previously linked to prostate cancer or other cancer types, which strengthens the result of this NGS analysis.

**Table 1:** NGS analysis of miRNAs in urinary EVs from 70 patients with different ISUP grades: 23 with Grade 1, 23 with Grade 2 and 24 with Grade 3. Adj. adjusted.

Grade 3 versus Grade 1			
**miRNA**	**Fold change**	***P*** **value**	**Adj.** ***P*** **value**
miR-1290	2.82	5.47E-05	0.015
miR-320b	1.62	8.89E-05	0.015
miR-1246	2.62	1.95E-04	0.021
miR-204-3p	1.53	4.62E-04	0.037
miR-143-3p	2.67	7.10E-04	0.046
**Grade 2 versus Grade 1**			
miR-155-5p	−2.73	1.31E-05	0.004
**Grade 3 versus Grade 2**			
miR-320a-3p	1.38	5.44E-05	0.017
miR-186-5p	−1.28	1.85E-04	0.022
miR-30e-5p	−1.26	2.05E-04	0.022
**Grade 3 versus Grades 1** + **2**			
miR-320a-3p	1.31	1.24E-04	0.043

**Fig. 3:**
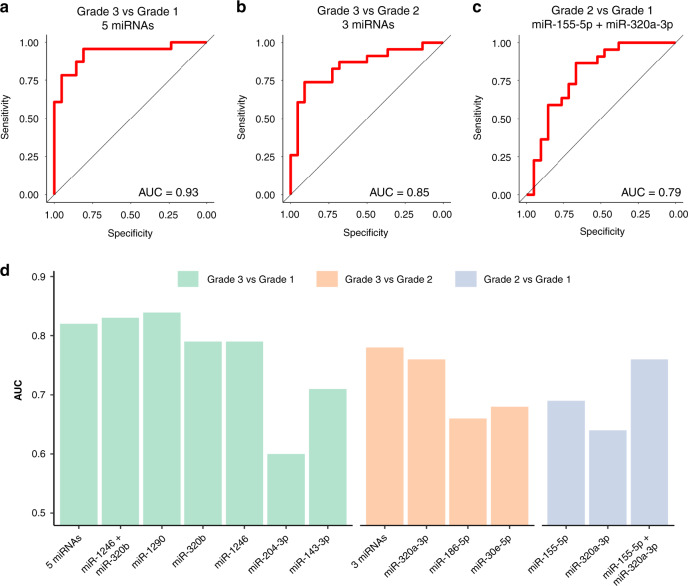
Evaluation of the predictive power of the miRNAs identified by NGS. **a**–**c** ROC curves and AUC values for the best models generated using the NGS data for each one to one-grade comparison. Five miRNAs: miR-1290, miR-1246, miR-320b, miR-204-3p and miR-143-3p. Three miRNAs: miR-320a-3p, miR-30e and miR-186. **d** Repeated fivefold cross-validation was used to estimate the behaviour of the miRNAs in an independent cohort. The graph shows, for each comparison, the calculated AUC of each individual miRNA and the best combinations of several miRNAs.

To further explore the usefulness of these miRNAs to discriminate between the different grades, we used their expression, measured in raw counts per million (cpm), to construct general linear models that could predict the grade of a certain sample based on the expression of several miRNAs. The efficiency of each model was estimated by measuring the AUC of ROC curves. When the five miRNAs differentially expressed between Grades 1 and 3 (miR-1290, miR-320b, miR-1246, miR-204-3p and miR-143-3p) were used, an AUC value of 0.93 was obtained (Fig. 3a). To simplify the model, we used a Lasso approach to determine which miRNAs were the best predictors. A combination of miR-1246 and miR-320b showed an AUC of 0.88. The model using the three miRNAs that were differentially expressed between Grades 3 and 2 (miR-320a-3p, miR-186-5p and miR-30e-5p) was able to predict the grade of a patient with an AUC of 0.85 (Fig. 3b). A model including the only miRNA significantly different between Grades 2 and 1, miR-155-5p, could predict the patient grade with an AUC 0.73. We noticed that the addition of miR-320a-3p, which is slightly decreased in Grade 2 compared to 1 (*P* value 0.02, not adjusted for multiple testing), improved the efficiency of this model to an AUC of 0.79 (Fig. 3c). Finally, models were also created to discriminate between one of the grades and the other two together. When comparing Grade 1 against Grades 2 and 3, the best efficiency was obtained with miR-155-5p alone (AUC 0.70) (Supplementary Fig. 3A). The best model to differentiate between Grades 1 and 2 together and Grade 3 was obtained with a combination of miR-1290, miR-1246, miR-204-3p and miR-320a-3p (AUC 0.87, Supplementary Fig. 3B). These results suggest these miRNAs could be useful to stratify patients in different grades. Moreover, their efficiency to distinguish between Grades 3 and 2 seem to be higher than between Grades 2 and 1.

### Cross-validation analysis of NGS data

A repeated fivefold cross-validation analysis was performed to get an estimation of how these nine miRNAs (Table 1) would behave in an independent dataset (Fig. 3d). In the comparison between Grades 3 and 1, the model with the five miRNAs differentially expressed (miR-1290, miR-1246, miR-320b, miR-204-3p and miR-143-3p) had an AUC of 0.82, while the combination previously selected by Lasso, miR-1246 and miR-320b, showed and AUC of 0.83. Remarkably, miR-1290 alone was able to differentiate between these grades with an AUC of 0.84, suggesting that it is a very robust biomarker for this comparison. When comparing Grades 3 and 2, the combination of the 3 miRNAs (miR-320a-3p, miR-30e and miR-186) showed an AUC of 0.78, and miR-320a-3p had the best efficiency when tried alone (AUC 0.76). The only miRNA differentially expressed between Grades 2 and 1, miR-155-5p, showed an AUC of 0.69, but adding miR-320a-3p to the model improved the result to an AUC of 0.76.

Using these models and the previous models performed using the whole dataset, we proceeded to the RT-qPCR analysis of seven miRNAs: miR-1290, miR-1246, miR-320b, miR-155-5p, miR-320a-3p, miR-186-5p and miR-30e-5p, in an independent patient cohort. We decided to exclude miR-204-3p and miR-143-3p because these miRNAs did not show a good predictive value.

### RT-qPCR analysis of selected miRNAs

Urinary small EVs isolated from a new cohort of 60 patients (20 from each grade, Supplementary Table 2) were measured by RT-qPCR to further analyse the selected miRNAs. We used an amount of EVs equivalent to 2–3 µg total protein. EV samples were treated with Proteinase K and RNase prior to RNA extraction to eliminate proteins and RNA outside EVs. Besides, the RNA isolation protocol included a DNase treatment to avoid DNA contamination. Several approaches have been suggested for the normalisation of urinary EV analysis [57], but an optimal method has not yet been identified for RT-qPCR results [58]. Based on the NGS data, we decided to use miR-99b-5p for normalisation because it was abundant and unchanged among the samples. This miRNA showed a good expression level (an average of 3590 cpm), no differential expression between groups (adjusted *P* value >0.99 in all five comparisons) and low variability within grades. Two samples from Grades 1 and 3 were discarded because all the miRNAs show very high *Cq* values.

**Fig. 4:**
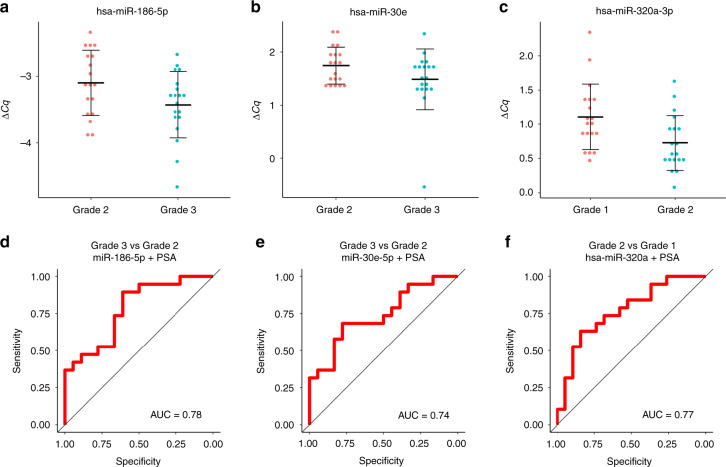
miRNAs in urinary EVs from a new cohort of 60 prostate cancer patients (20 per ISUP grade) were analysed using RT-qPCR. **a**–**c** Comparison of the expression of miR-186-5p and miR-30e-5p between Grades 3 and 2, and miR-320a-3p between Grades 2 and 1. *P* values: **a**—0.049, **b**—0.1, **c**—0.012. **d**–**f** ROC curves and AUC of the predictive models generated with miRNAs and PSA levels.

Some of the analysed miRNAs showed in this second cohort a similar pattern as the NGS data (Supplementary Table 3 and Supplementary Fig. 4A–E). We found that miR-186-5p also was significantly decreased between Grades 3 and 2 (*P* value 0.049, Fig. 4a), and that miR-30e-5p showed a decrease in Grade 3 compared to Grade 2, although not statistically significant (*P* value 0.1, Fig. 4b). miR-320a-3p, which in the NGS stage had shown a significant difference between Grades 3 and 2 and good predictive power between Grades 2 and 1, showed in this cohort a significant decrease in Grade 2 compared to Grade 1 (*P* value 0.012, Fig. 4c). These three miRNAs were detected in at least 18 samples of each grade. Unfortunately, we could not analyse the expression of two of the miRNAs which had shown a very good predictive value in the first stage, miR-1290 and miR-155-5p, because the RT-qPCR signal was too low to be measured accurately. The RT-qPCR results for miR-1246 and miR-320b for Grade 3 vs. Grade 1, and miR-320a-3p for Grade 3 vs. Grade 2 did not reflect the NGS results (Supplementary Table 3).

Finally, we used the RT-qPCR data to construct predictive linear models to estimate the ability of the 3 miRNAs that had shown a similar pattern in the NGS and RT-qPCR cohorts (miR-320a-3p, miR-186-5p and miR-30e-5p) to discriminate between the different grades. When analysed alone, these miRNAs did not show a good predictive power (Supplementary Fig. 4F–H). Then, we investigated whether adding the patients’ serum PSA value improved the predictions. In our samples, PSA alone showed a similar predictive power as the miRNAs in the comparison between Grades 3 and 2 (AUC 0.67). However, combining PSA with the expression of miR-186-5p or miR-30e-5p resulted in an improvement of the models, from an AUC value of 0.67 to 0.78 and from 0.68 to 0.74, respectively (Fig. 4d, e and Supplementary Fig. 4F, G). For the comparison between Grades 2 and 1, PSA alone was a bad predictor to distinguish between groups (AUC 0.61) and adding it to the model with miR-320a-3p resulted in a slight improvement from AUC 0.74 to 0.77 (Fig. 4f and Supplementary Fig. 4H). In conclusion, the RT-qPCR results were not so strong as the NGS results, but further suggest that miR-320a-3p and miR-186-5p can be good candidates for patient stratification based on their ISUP Grade, and that adding PSA to the models can enhance their predictive value.

## Discussion

Prostate cancer patients with Grade 1 can safely be followed up by AS. Patients with Grades 2 and 3 show different prognoses and it is possible that Grade 2 patients may benefit from this option too. In this study, NGS was used to identify novel miRNAs in small urinary EVs that could help to distinguish these grades. miRNAs derived from EVs have been used to stratify prostate cancer patients before [41, 42, 59], but previous studies have included patients with ISUP Grades 2 and 3 in the same group. Our results showed that 9 miRNAs had a significantly different expression in at least one of the possible comparisons between those three grades. A cross-validation analysis suggested that miR-1290 and miR-320a-3p were the most reliable miRNAs to differentiate between Grades 3 and 1 and Grades 3 and 2, respectively. Only 1 miRNA, miR-155-5p, was found to be differentially expressed between Grades 2 and 1.

Due to limitations in the amount of sample available, we used an additional cohort to strengthen the NGS results. This second analysis was done using RT-qPCR, a more clinically friendly method than NGS. The seven miRNAs that showed the best predictive value in the NGS analysis were further analysed by RT-qPCR. We found that the expression levels of miR-186-5p and miR-30e-5p in the comparison between Grade 3 and Grade 2, and miR-320a-3p for Grade 2 and Grade 1, showed similar results in both cohorts, thus making the identification of these miRNAs more reliable and increasing the potential of these miRNAs as prostate cancer biomarkers. The addition of PSA to some of the models increased the performance of these miRNAs to some extent.

Some of the more promising miRNAs identified by NGS, miR-1290 and miR-155-5p, were not detected with the RT-qPCR protocol used. In this study, we used sequential ultracentrifugation to isolate small EVs from urine. There are several comparative studies of methods to isolate urinary EVs for RNA analysis [60, 61]. These studies, which include ultracentrifugation, commercial isolation kits, hydrostatic filtration dialysis and affinity-based purification protocols, show that different isolation methods may lead to different yields and results. It would therefore be interesting to validate these results using other methods, preferentially methods that are easily translated into clinical labs.

We were not able to confirm by RT-qPCR the differences observed in NGS with miR-1246 and miR-320b (Supplementary Table 3). Future experimental optimisation of the PCR protocol may facilitate further validation studies. Moreover, the normalisation method is a key unresolved aspect of EV miRNA expression using RT-PCR. Several strategies have been proposed, including the global mean of all the studied miRNAs or the expression of one or more endogenous miRNAs or exogenous spike-in controls. In this study, we normalised using the endogenous miRNA miR-99b-5p based on its high expression and intra- and intergroup stability in the NGS data. However, this normalisation method may not be optimal for all the miRNAs that were analysed.

The identified miRNAs have been related to cancer/prostate cancer before. For example, the blood levels of miR-1290 have been previously proposed as a biomarker for early diagnosis of prostate cancer [62], while the expression of this miRNA in blood EVs could predict castration-resistant tumours [63]. It has also been reported that the levels of miR-1290 in cell-free urine are similar in healthy controls and prostate cancer patients [64]. However, cell-free and EV-derived miRNAs do not necessarily show the same expression profiles in urine [65]. miR-155-5p has been found to inhibit the migration of prostate cancer cells [66] and a potential target for antitumoral treatments [67]. It has also been proposed that downregulation of miR-186-5p promotes cell proliferation and invasion due to its effect on Twist1 [68], and low levels of miR-186-5p have also been linked to other tumours such as colorectal and esophageal cancer [69, 70]. Downregulation of miR-30e-5p has been related to prostate cancer through its regulatory effect over CTHRC1, a protein upregulated in prostate cancer patients [71]. This miRNA has been also proposed as a potential biomarker in other types of cancer such as non-small-cell lung tumours and squamous cell carcinoma [72, 73]. miRNAs of the miR-320 family have been found to be decreased in the serum of prostate cancer patients compared with patients with BPH [74]. It has been suggested that these miRNAs negatively regulate the Wnt/β-catenin pathway [75], whose activation promotes the progression of prostate tumours [76].

It is important to identify miRNAs that can stratify patients with Grades 2 and 3, as patients with both grades usually undergo radical treatment despite their different prognoses. Having a reliable method to distinguish between these grades could increase the number of Grade 2 patients who can benefit from AS, improving their quality of life. Establishing the correct GS is complicated and requires an experienced pathologist, and even then there may be a significant variability within experts [77]. In addition, the biopsy pathological classification is often down- or upregulated when the prostate tissue is analysed after surgery. The analysis of the EV molecular content in liquid biopsies could therefore be used to complement the tissue-based diagnosis and help to obtain a more reliable diagnosis. Besides, monitoring of AS patients involves the collection of tissue biopsies, which may have side effects. Urine samples can be obtained frequently in a non-invasive way, which will facilitate AS monitoring.

EVs in urine originate from different organs of the genitourinary system and are expected to be released by different mechanisms [78]. Recent research has shown that the size of these vesicles overlaps to some extent, making it difficult to separate the two vesicular populations based on their size. Our results show that the protein profile and size distribution of the pellets obtained at 10,000×*g* (large EVs) and 100,000×*g* (small EVs) are different and that the 100,000×*g* pellet is enriched in markers associated with vesicles derived from MVBs [79]. Even if from a biomarker perspective both pellets could have been studied together, we decided to focus on the 100,000×*g* pellet to analyse a more homogenous population of vesicles. Besides, most of the previous studies searching for biomarkers in EVs have used the 100,000×*g* pellet and it makes easier to compare our results to different studies. Finally, NTA analysis showed that the number of particles was relatively low in the 10,000×*g* pellet, which made it difficult to analyse the miRNA content of this fraction.

In conclusion, NGS revealed several promising miRNAs for the stratification of prostate cancer patients with ISUP Grades 1, 2 and 3. Additional RT-qPCR analysis supports some of the results, but further studies are needed to confirm the potential of the miRNAs identified by NGS as prostate cancer biomarkers. Besides, additional molecular or clinical parameters could be added to these miRNAs to improve the separation of the patient groups. Combining traditional tissue biopsies with liquid biopsies could increase the quality of life of patients under AS, and these miRNAs in urinary EVs are promising candidates that deserve further research.

## Supplementary information

SupplementaryTablesandFigures

Dataset1
