# Supplementary material for: Potential of miRNAs in urinary extracellular vesicles for management of active surveillance in prostate cancer patients
Source: Br J Cancer. 2021 Nov 22;126(3):492–501. doi: 10.1038/s41416-021-01598-1 (PMC8810884; doi:10.1038/s41416-021-01598-1)
Supplement: Supplementary file 1 — SupplementaryTablesandFigures [file 41416_2021_1598_MOESM1_ESM.pdf]

Supplementary table 1

Total information			
	**Grade 1**	**Grade 2**	**Grade 3**
**Number of patients**	23	23	23
**Mean age (years)**	65.5 ± 5.9	64.7 ± 5.2	66 ± 6
**Min age (years)**	48	54	55
**Max age (years)**	73	73	77
**Mean PSA (ng/ml)**	5.6 ± 2.1	6.3 ± 1.9	6.7 ± 3.1
**Min PSA (ng/ml)**	2.4	2.3	1.1
**Max PSA (ng/ml)**	9.3	9.6	15
**PSA (0-4 ng/ml)**	5	2	3
**PSA (4-10 ng/ml)**	18	21	19
**PSA (>10 ng/ml)**	0	0	2

Individual information					
**Patient number**	**ISUP grade**	**Clinical stage**	**PSA (ng/ml)**	**Age (years)**	**Urisys**
1	1	T1c	8	73	pH5
2	1	T1c	7.1	59	pH6
3	1	T1c	8.8	72	pH6, glu 100
4	1	T1c	3.5	68	pH5
5	1	T1c	4.9	67	pH7
6	1	T1c	2.4	71	pH6
7	1	T1c	2.7	66	pH5
8	1	T2	5	60	pH5
9	1	T2	4.8	57	pH6
10	1	T1c	4.6	72	pH5
11	1	T2	3.1	77	pH5, ery 25
12	1	T1c	8.5	71	pH6
13	1	T2	6.5	66	pH5, ket 15
14	1	T2	4.3	55	pH7, ery 50
15	1	T1c	5.4	68	pH5, ket 15
16	1	T1c	4.5	62	pH5
17	1	T1c	9.3	71	pH6
18	1	T1c	7.4	64	pH6
19	1	T1c	7.7	64	pH6
20	1	T1c	6.5	65	pH6
21	1	T2	2.9	48	pH6
22	2	T2	5.4	68	pH5
23	2	T2c	9.2	64	pH7
24	2	T2	2.3	66	pH6
25	2	T1c	5.4	54	pH7
26	2	T2	4.4	73	pH5, glu 1000, pro 150
27	2	T1c	6.1	65	pH5, ery 25

28	2	T2	6.4	66	pH8
29	2	T1c	2.7	57	pH6
30	2	T1c	6	60	pH6
31	2	T2	7.7	61	pH7
32	2	T2	8.9	70	pH5
33	2	T2	7.2	62	pH5
34	2	T1c	4.8	64	pH6
35	2	T2	5.2	70	pH8
36	2	T1c	8.4	69	pH5
37	2	T2	8	62	pH5, glu 300
38	2	T1c	6.1	70	pH5
39	2	T2	8	71	pH5
40	2	T1c	4.4	55	pH6
41	2	T1c	5.6	66	pH8
42	2	T2	6.6	65	pH6
43	2	T2	9.6	69	pH5
44	2	T2	6.2	60	pH6
45	3	T1c	5.2	69	pH5
46	3	T2	6.9	70	pH5
47	3	T2	7.9	65	pH5, ery 50
48	3	T1c	5.9	68	pH6, ery 10
49	3	T2	5.3	61	pH6
50	3	T1c	5.5	69	pH7, leu 25
51	3	T2	5.6	65	pH8
52	3	T3b	9	77	pH6
53	3	T2	1.1	55	pH5 ket 15 leu 25 pro 25
54	3	T1c	8.3	74	pH7
55	3	T1c	6.1	58	pH7
56	3	T2a	5.4	69	pH5
57	3	T2	7.5	73	pH5, ery 25
58	3	T1c	4.4	66	pH6
59	3	T2	4.7	57	pH5
60	3	T2	6.1	63	pH5
61	3	T2	8	65	pH7
62	3	T2	1.6	67	pH5
63	3	T1c	13	72	pH5
64	3	T1c	10	71	pH6
65	3	T1c	6.2	56	pH6
66	3	T3a	8	72	pH5
67	3	T2	3.1	62	pH5
68	3	T2	15	60	pH5

Supplementary table 1: NGS patient cohort. Summary of the preocuperative ISUP grade, age and PSA level of the cohort used in the NGS analysis and individualized patient information. PSA (prostate-specific antigen) is shown in ng/ml. The Urisys column shows the pH and the parameters that showed values above low levels: leu. (leukocytes, 25 cells/μl), pro. (protein, 25 mg/dl), glu. (glucose, 50 mg/dl), ket. (ketone, 5 mg/dl), ery. (erythrocytes, 10 cells/μl).

Supplementary table 2

Total information			
	**Grade 1**	**Grade 2**	**Grade 3**
**Number of patients**	20	20	20
**Mean age (years)**	64.5 ± 5.8	66 ± 6.2	66.6 ± 7.6
**Min age (years)**	52	54	50
**Max age (years)**	74	75	77
**Mean PSA (ng/ml)**	7.2 ± 3.7	9.2 ± 7.4	14.4 ± 12.1
**Min PSA (ng/ml)**	2.8	3.9	4.4
**Max PSA (ng/ml)**	18	39	50
**PSA (0-4 ng/ml)**	3	1	0
**PSA (4-10 ng/ml)**	14	17	13
**PSA (>10 ng/ml)**	3	2	7

Individual information					
**Patient number**	**ISUP grade**	**Clinical stage**	**PSA (ng/ml)**	**Age (years)**	**Urisys**
1	1	T1c	5.1	65	pH6
2	1	T2	10	59	pH8
3	1	T2	6	64	pH5
4	1	T1c	2.8	69	pH7, leu 100
5	1	T1c	4.7	58	pH5
6	1	T1c	7	66	pH7
7	1	T1c	6.6	52	pH6
8	1	T2	7.2	62	pH5, glu 1000, ket 15
9	1	T1c	5.8	66	pH5
10	1	T1c	18	74	pH5
11	1	T1c	8.9	71	pH8
12	1	T1c	1.2	67	pH6
13	1	T1c	12	63	pH5
14	1	T1c	8.5	67	pH8
15	1	T2b	7.2	62	pH5, glu 1000
16	1	T1c	5.7	67	pH6
17	1	T1c	4	73	pH6
18	1	T1c	5.4	66	pH6
19	1	T1c	12	54	pH5
20	2	T2b	9.5	62	pH5
21	2	T1c	8.1	64	pH7
22	2	T1c	5.3	55	pH7
23	2	T1c	8	73	pH5
24	2	T2b	6.6	67	pH5
25	2	T1c	10	71	pH6
26	2	T3a	7.7	67	pH6
27	2	T1c	15	67	pH6, leu 500, ery 250

28	2	T1c	7.2	62	pH6
29	2	T3a	9.2	75	pH5, ket 15
30	2	T1c	3.9	57	pH6
31	2	T1c	6.1	67	pH5
32	2	T2b	6.3	69	pH6
33	2	T3a	6.7	62	pH6
34	2	T2b	9.5	71	pH6
35	2	T1c	8.6	75	pH5, glu 1000, ket 15
36	2	T1c	5.1	54	pH5
37	2	T2b	5.9	64	pH5
38	2	T1c	5.7	66	pH6
39	2	T2	39	73	pH7, leu 500
40	3	T3	6	61	pH6
41	3	T1c	7.4	70	pH5, glu 1000, ket 15
42	3	T1c	7.7	77	pH5
43	3	T1c	50	72	pH5
44	3	T3	9.8	67	pH5
45	3	T2	4.4	78	pH7
46	3	T1c	8.1	68	pH5, ket 15
47	3	T2	5.7	64	pH5, ket 15, ery 25
48	3	T3	9.9	61	pH6
49	3	T1c	22	50	pH8
50	3	T1c	5.6	70	pH5
51	3	T3b	11	76	pH6
52	3	T2	7.7	69	pH5
53	3	T3a	14	70	pH5
54	3	T2	7.5	60	pH7
55	3	T2	8.4	70	pH6
56	3	T2	27	66	pH6
57	3	T2	39	51	pH8
58	3	T2	21	66	pH6

Supplementary table 2: RT-qPCR patient cohort. Summary of the preocuperative ISUP grade, age and PSA level of the cohort used in the RT-qPCR analysis and individualized patient information. PSA (prostate-specific antigen) is shown in ng/ml. The Urisys column shows the pH and the parameters that showed values above low levels: leu. (leukocytes, 25 cells/µl), pro. (protein, 25 mg/dl), glu. (glucose, 50 mg/dl), ket. (ketone, 5 mg/dl), ery. (erythrocytes, 10 cells/µl).

Grade 3 vs grade 1				
**miRNA**	**NGS Fold change**	**NGS p-value**	**RT-qPCR Fold change**	**RT-qPCR p-value**
miR-1290	2.82	0.015	nd	nd
miR-320b	1.62	0.015	-1.35	0.035
miR-1246	2.62	0.021	-1.1	0.84
**Grade 2 vs grade 1**				
**miRNA**	**NGS Fold change**	**NGS p-value**	**RT-qPCR Fold change**	**RT-qPCR p-value**
miR-155-5p	-2.73	0.004	nd	nd
miR-320a-3p	1.90	0.360	1.3	0.012
**Grade 3 vs grade 2**				
**miRNA**	**NGS Fold change**	**NGS p-value**	**RT-qPCR Fold change**	**RT-qPCR p-value**
miR-320a-3p	1.38	0.017	-1.01	0.92
miR-186-5p	-1.28	0.022	-1.26	0.049
miR-30e-5p	-1.26	0.022	-1.2	0.1

Supplementary table 3: Seven miRNAs selected according to the NGS results were analyzed by RT-qPCR in an independent cohort. The table shows a comparison of the results obtained by NGS and RT-qPCR. Highlighted miRNAs showed the same pattern in both cohorts. nd = not detected.

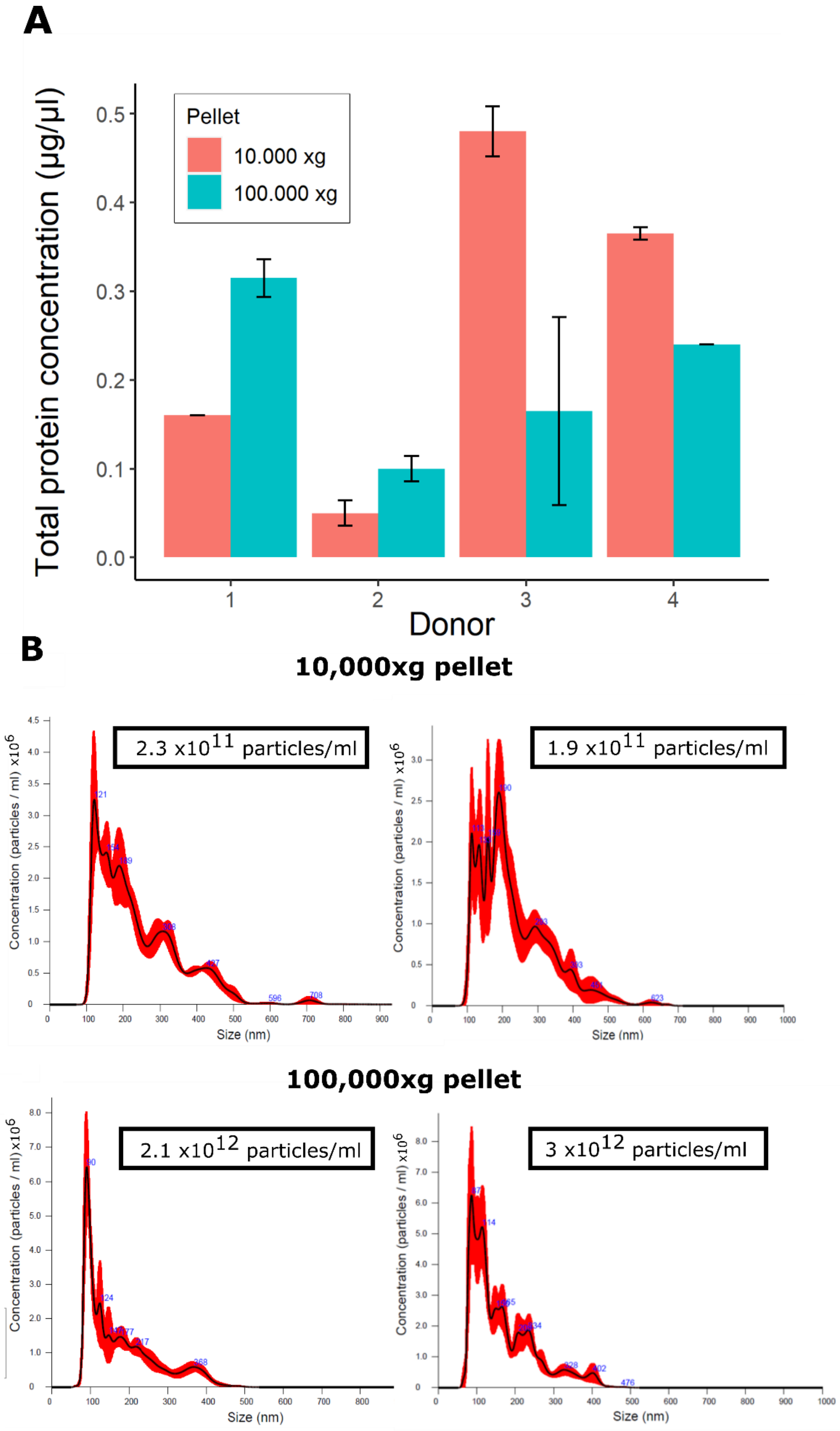
A
Total protein concentration (µg/µl)
Pellet
10.000 xg
100.000 xg
0.0
0.1
0.2
0.3
0.4
0.5
1
2
3
4
Donor
B
10,000xg pellet
2.3 x10^11 particles/ml
1.9 x10^11 particles/ml
Concentration (particles / ml) x10^6
Size (nm)
100,000xg pellet
2.1 x10^12 particles/ml
3 x10^12 particles/ml
Concentration (particles / ml) x10^6
Size (nm)

Supplementary figure 1: Characterization of the 10,000 xg and 100,000 xg pellets. A: The 10,000 xg and 100,000 xg pellet were isolated from urine samples of 4 healthy donors. The concentration of total protein in each pellet was measured using a Qubit 3 fluorometer. Standard deviation between duplicates is shown. B: Nanosight analysis of particles in the 10,000 xg pellet and 100,000 xg pellet from the urine of 2 healthy donors. The mean sizes were 241.4 nm and 231.7 nm for the 10,000 xg pellets and 177.8 nm and 167.3 nm for the 100,000 xg pellet. The mode sizes were 120.8 nm and 189.4 nm for the 10,000 xg pellets and 89.8 nm and 86.0 nm for the 100,000 xg pellet.

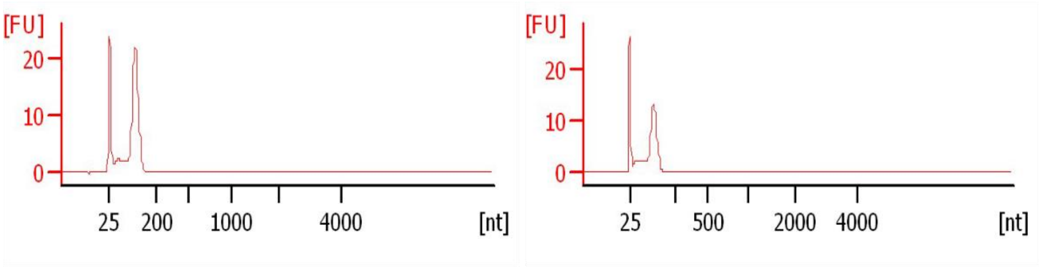

Supplementary figure 2: Typical Bioanalyzer profile of the small RNA samples used in the NGS analysis. EVs were treated with Proteinase K and RNAase to remove extravesicular molecules, and then the small RNAs were isolated using the miRNeasy Mini kit.

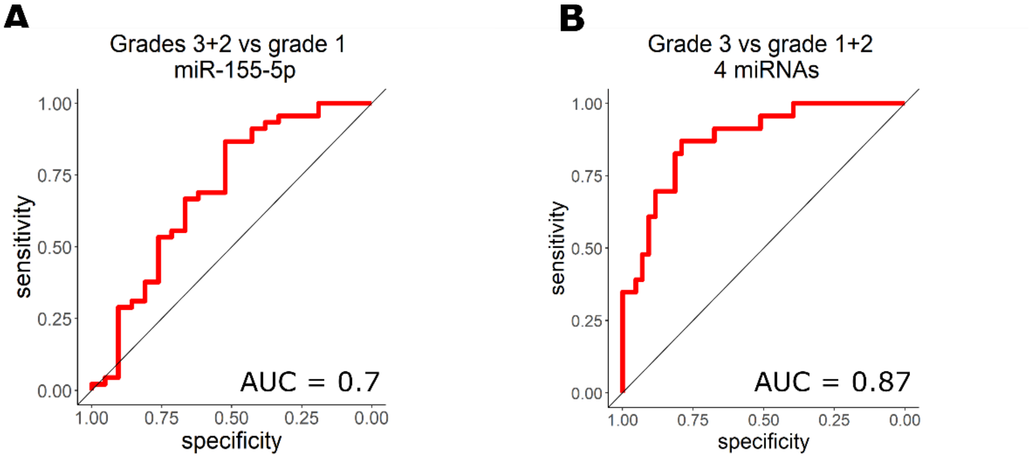

Supplementary figure 3: Evaluation of the predictive power of the miRNAs identified by NGS in comparisons of two grades together against the other one. A: ROC curve and AUC value of the best miRNA for the comparison of grade 1 to grades 2 and 3 together. B: ROC curve and AUC value of the 4 miRNAs with p-value lower than 0.1 for the comparison grade 3 against grades 1 and 2 together.

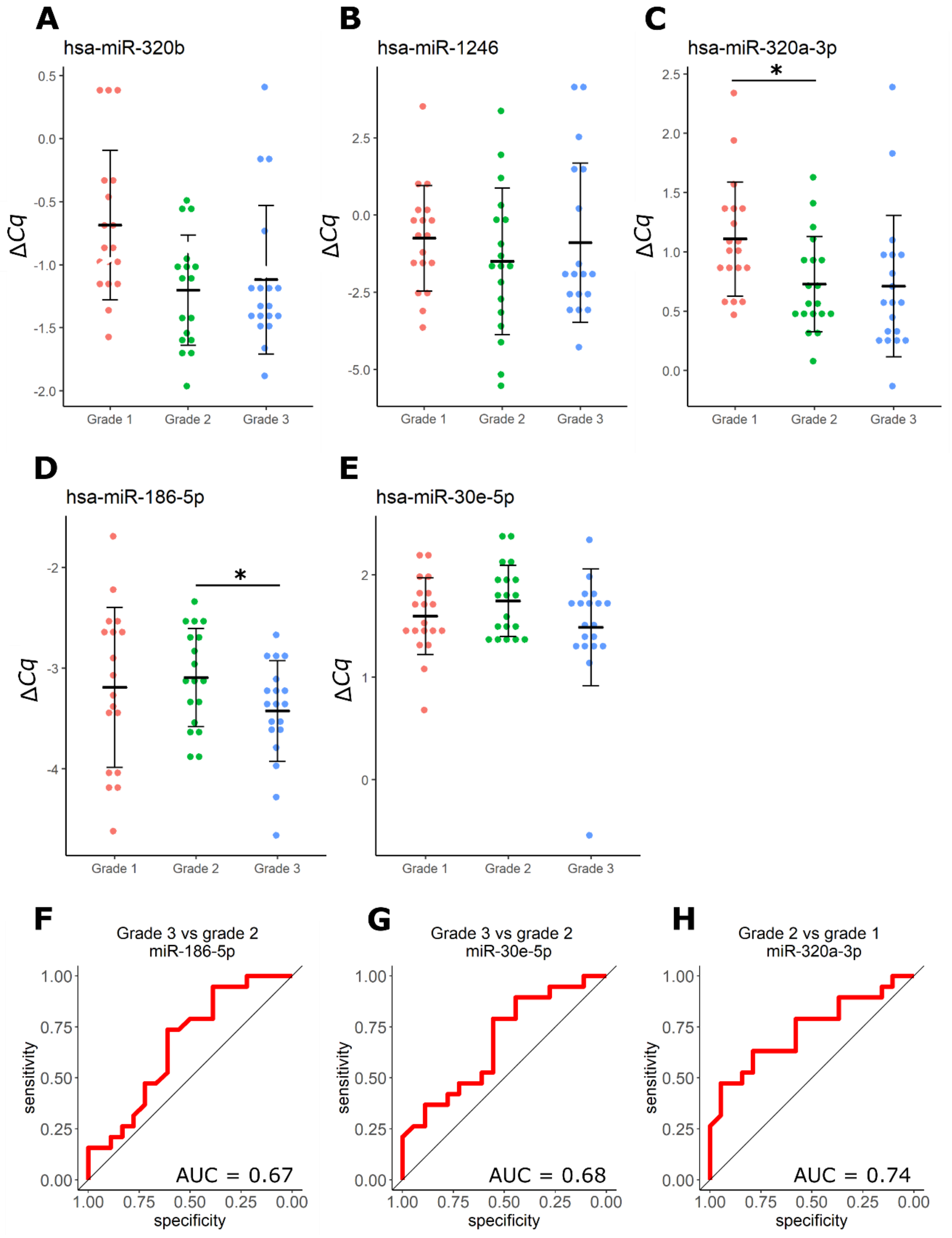
A
hsa-miR-320b
B
hsa-miR-1246
C
hsa-miR-320a-3p
D
hsa-miR-186-5p
E
hsa-miR-30e-5p
ΔCq
Grade 1
Grade 2
Grade 3
*
F
Grade 3 vs grade 2
miR-186-5p
AUC = 0.67
G
Grade 3 vs grade 2
miR-30e-5p
AUC = 0.68
H
Grade 2 vs grade 1
miR-320a-3p
AUC = 0.74
sensitivity
specificity

Supplementary figure 4: A-E: RT-qPCR results for the 3 different grades for miRNAs miR-320b, miR-1246, miR-320a-3p, miR-186-5p and miR-30e-5p. Asterisks signal the grades were the miRNA expression was significantly different (p-value < 0.05). F-H: ROC curves and AUC of the predictive models generated with RT-qPCR data for miR-186-5p, miR-30e-5p and miR-320a-3p.
